# COVID-19 admission risk tools should include multiethnic age structures, multimorbidity and deprivation metrics for air pollution, household overcrowding, housing quality and adult skills

**DOI:** 10.1136/bmjresp-2021-000951

**Published:** 2021-08-09

**Authors:** Marina A Soltan, Justin Varney, Benjamin Sutton, Colin R Melville, Sebastian T Lugg, Dhruv Parekh, Will Carroll, Davinder P Dosanjh, David R Thickett

**Affiliations:** 1 Birmingham Acute Care Research Group, Institute of Inflammation and Ageing, University of Birmingham, Birmingham, UK; 2 University Hospitals Birmingham Foundation NHS Trust, Birmingham, UK; 3 Health Inequalities Research Unit, England, United Kingdom, Great Britain; 4 Birmingham City Council, Birmingham, UK; 5 Birmingham Lung Research Unit, Birmingham, UK; 6 The University of Manchester Faculty of Medical and Human Sciences, Manchester, UK; 7 University Hospitals North Midlands, Stoke on Trent, UK; 8 College of Medical and Dental Sciences, University of Birmingham, Birmingham, UK

**Keywords:** COVID-19, pneumonia, respiratory infection, viral infection

## Abstract

**Background:**

Ethnic minorities account for 34% of critically ill patients with COVID-19 despite constituting 14% of the UK population. Internationally, researchers have called for studies to understand deterioration risk factors to inform clinical risk tool development.

**Methods:**

Multicentre cohort study of hospitalised patients with COVID-19 (n=3671) exploring determinants of health, including Index of Multiple Deprivation (IMD) subdomains, as risk factors for presentation, deterioration and mortality by ethnicity. Receiver operator characteristics were plotted for CURB65 and ISARIC4C by ethnicity and area under the curve (AUC) calculated.

**Results:**

Ethnic minorities were hospitalised with higher Charlson Comorbidity Scores than age, sex and deprivation matched controls and from the most deprived quintile of at least one IMD subdomain: indoor living environment (LE), outdoor LE, adult skills, wider barriers to housing and services. Admission from the most deprived quintile of these deprivation forms was associated with multilobar pneumonia on presentation and ICU admission. AUC did not exceed 0.7 for CURB65 or ISARIC4C among any ethnicity except ISARIC4C among Indian patients (0.83, 95% CI 0.73 to 0.93). Ethnic minorities presenting with pneumonia and low CURB65 (0–1) had higher mortality than White patients (22.6% vs 9.4%; p<0.001); Africans were at highest risk (38.5%; p=0.006), followed by Caribbean (26.7%; p=0.008), Indian (23.1%; p=0.007) and Pakistani (21.2%; p=0.004).

**Conclusions:**

Ethnic minorities exhibit higher multimorbidity despite younger age structures and disproportionate exposure to unscored risk factors including obesity and deprivation. Household overcrowding, air pollution, housing quality and adult skills deprivation are associated with multilobar pneumonia on presentation and ICU admission which are mortality risk factors. Risk tools need to reflect risks predominantly affecting ethnic minorities.

**Key messages**

- To what extent are determinants for health, including Index of Multiple Deprivation subdomains with indicators for household overcrowding, housing quality, air pollution and adult skills deprivation, risk factors for presentation with multilobar pneumonia, Intensive Therapy Unit (ICU) admission and outcomes among individual ethnic minority groups hospitalised with COVID-19?

- Ethnic minorities exhibit higher multimorbidity despite younger age structures and disproportionate exposure to unscored risk factors including obesity and hospitalisation from the most deprived quintile for household overcrowding, air pollution, housing quality and adult skills; current admission risk stratification tools do not account for socio-environmental risk factors.

- Understanding the risk factors for presentation with multilobar pneumonia, ICU admission and mortality among individual ethnic minority groups is essential for the identification of patients at risk of deterioration, supporting triage to the appropriate level of care and informing the development of clinical risk stratification tools.

## Introduction

Ethnic minorities account for 34% of critically ill patients with SARS-CoV-2 infection (COVID-19) despite constituting 14% of the UK population according to the UK Office for National Statistics (ONS).^1^ Effective triage at the point of admission to hospital is required to ensure that patients from all ethnic groups are risk stratified to the appropriate level of care. Internationally, researchers have called for studies to understand deterioration and mortality risk factors to inform clinical risk tool development.^2^

Diagnostic and prognostication models are valuable for risk stratification at the point of admission; more than 232 models for COVID-19 have been put forward by the academic community.^3^ However, critical appraisal of these models has identified that candidate models are poorly reported, at high risk of bias and their risk stratification performance among individual ethnic groups has not been reported.^4^ Moreover, most of these models are based on retrospective studies and prospective studies are scarce. Yildiz *et al*^5^ recently prospectively compared and validated ISARIC4C, CURB65, NEWS2 and COVID-GRAM and showed that CURB65 and ISARIC4C were useful predictors of mortality in patients with COVID-19. However, they did not study the impact of ethnicity. It is therefore unclear how well these proposed models perform in practice to risk stratify individual ethnic minority groups and whether models sufficiently account for biological and socioenvironmental risk factors to which ethnic minorities are predominantly predisposed.

We aimed to address this knowledge gap by exploring determinants of health, including Index of Multiple Deprivation (IMD) subdomains, as risk factors for presentation, deterioration and mortality by ethnicity and by evaluating the performance of two widely used prognostic models, CURB65 and ISARIC4C, among hospitalised patients diagnosed with COVID-19 by ethnicity.^6 7^

Clinical training has reinforced that the unmodifiable risk factor of age predisposes to adverse outcomes with little regard to the epidemiological variation in the age structures of different ethnic groups, also known as multiethnic age structures. Ethnic minorities have younger age structures that predispose to a lower risk score using current risk stratification tools.^8^ Furthermore, ethnic minorities more frequently exhibit obesity and higher multimorbidity despite presenting younger yet this risk profile is not considered in current risk stratification tools.

Moreover, ethnic minorities are more likely than White patients to be hospitalised with COVID-19 from the most deprived IMD areas.^9^ UK data published by the Office for National Statistics (ONS) shows higher age-standardised mortality rates for COVID-19 in the most deprived IMD areas (3.1 deaths per 100 000 patients) compared with the least deprived (1.4 deaths per 100 000) between 1 March 2020 and 31 July 2020.^10^ However, studies have not yet explored individual IMD subdomains as risk factors for presentation with multilobar pneumonia, intensive care unit (ICU) admission and completed hospitalised episode outcomes. The IMD incorporates seven weighted deprivation domains: income, employment, health, crime, barriers to housing and services (BHS), living environment (LE) and education, skills and training (EST).^11^ BHS, LE and EST domains each have two subdomains. BHS subdomains include: (A) geographical barriers, an indicator of proximity to local services and (B) wider BHS that contains an indicator for household overcrowding. LE subdomains include: (a) indoor LE, which has an indicator for housing quality and (B) outdoor LE, which has an indicator for air pollution. EST subdomains include: (A) children and younger people’s education attainment and (B) adult skills that contains indicators for adult qualifications and English language proficiency.^11^

Understanding these biological, demographic and socioenvironmental risk factors is invaluable when it comes to evaluating the reliability of current risk stratification tools and informing the development of stratification tools that reflect risk factors to which ethnic minorities are potentially disproportionately predisposed.

## Methods

### Design and setting

A multicentre cohort study of hospitalised patients with COVID-19 (n=3671) was performed to explore social determinants of health, including IMD subdomains, as risk factors for presentation with multilobar pneumonia, ICU admission and hospitalised outcomes.

### Patient population

COVID-19 positive patients (>16 years old) with a confirmed PCR-positive analysis of a combined nose and throat swab in accordance with Public Health England guidance from four hospitals across the West Midlands, University Hospitals of Birmingham, between 1 February 2020 and 1 September 2020 were included.^12^

### Patient management

See online supplemental 1.

10.1136/bmjresp-2021-000951.supp1 Supplementary data

### Data collection and scoring analysis

Hospital informatics data included: demographics (ethnicity, age and IMD), admission details, comorbidities, clinical metrics (observations and blood tests), imaging, ICU admission details and hospitalised episode outcomes. Chest X-rays were reported by radiologists within 12 hours of being undertaken.

### Index of Multiple Deprivation

IMD domains and subdomains are detailed above. The IMD categorises deprivation metrics by postcode. Detailed descriptions of IMD metrics are published by the UK Ministry of Housing, Communities and Local Government.^13^

### Charlson Comorbidity Index (CCI)

CCI is a validated tool quantifying comorbidity burden and corresponding 1-year mortality risk.^14^

### CURB65 and ISARIC4C

Characteristics of studies describing CURB65 and ISARIC4C mortality models^6 15 16^ are described in online supplemental 2.

### Statistical analysis

Baseline characteristics were presented as mean and SD for continuous variables and median and IQR for non-parametric data. Normality was assessed by Shapiro-Wilk test. For categorical and ordinal variables with non-parametric distribution, Fisher’s exact test and Mann-Whitney U test were used respectively for comparisons between two groups. Age-adjusted and sex-adjusted mortality were calculated by logistic regression analyses. Multivariate analysis to predict mortality was performed using stepwise logistic regression with conservative criteria for entry or exit from the model of 0.1. Variables listed in online supplemental 3 were included in multivariate analysis. The Hosmer and Lemeshow goodness-of-fit test was performed to evaluate logistic regression model adequacy. Matched case–control analyses (1:1) using IBM SPSS V.24 were implemented to explore underlying multimorbidity among ethnic minorities; controls were White patients matched by age, gender and deprivation subdomains. Performance of the CURB65 and ISARIC 4C tools among individual ethnic groups were assessed using receiver operating characteristic curves Area Under the Receiver Operator Curve (AUROC). Statistical analyses were carried out using SPSS V.24.

## Results

### Included participants

A total of 3671 consecutive patients were assessed for inclusion. Online supplemental 4 shows the Consolidated Standards of Reporting Trials diagram.

### Study population

#### Age and sex

The study population is outlined in table 1. Males (54.8%) were hospitalised more than females (45.2%). The median age of all patients was 76.0 (24.0) years. Ethnic minorities were more likely to present age <65 years (OR 4.85 (95% CI 4.02 to 5.84); p<0.001) than White patients. Caribbean and White groups presented older (median age >65 years), while Indian, Pakistani, African, Chinese and Bangladeshi groups presented younger (median age <65 years); this is consistent with UK population age structures.^8^

**Table 1:** A table showing patient characteristics including: age, gender, ethnicity, ICU admission, mortality and discharge

Participant Characteristics								
	All study COVID-19 positive patients median age (IQR)	All COVID-19 positive patients N (% of column total)	COVID-19 positive patients with radiological changes of pneumonia N (% of row total)	COVID-19 positive patients with radiological changes of multilobar pneumonia N (% of row total)	COVID-19-positive patients without radiological changes of pneumonia N (% of row total)	ICU admission N (% of row total)	Discharge N (% of row total)	Mortality N (% of row total)
N		2646	1667 (63.0)	1307 (49.4)	979 (37.0)	310 (11.7)	1771 (66.9)	875 (33.1)
Age, median (IQR)		76.0 (24.0)	70.8 (16.5)	69.4 (16.6)	73.7 (17.7)	58.5 (12.5)	68.8 (18.0)	78.1 (12.8)
Gender								
Male	73.0 (24.0)	**1449** (54.8)	970 (66.9)	775 (53.5)	479 (33.1)	220 (15.2)	921 (63.6)	528 (36.4)
Female	79.0 (23.0)	**1197** (45.2)	697 (58.2)	532 (44.4)	500 (41.8)	90 (7.5)	802 (67.0)	347 (29.0)
Ethnicity								
White	79.0 (19)	**1917** (72.4)	1123 (58.6)	831 (43.3)	794 (41.4)	161 (8.4)	1242 (64.8)	675 (35.2)
Indian	63.0 (23.5)	**93** (3.5)	77 (82.8)	66 (71.0)	16 (17.2)	21 (22.6)	67 (72.0)	26 (28.0)
Pakistani	62.0 (29.0)	**326** (12.3)	245 (75.2)	216 (66.3)	81 (24.8)	66 (20.2)	227 (69.6)	99 (30.4)
Caribbean	73.0 (28.0)	**105** (4.0)	69 (65.7)	58 (55.2)	36 (34.3)	10 (9.5)	73 (69.5)	32 (30.5)
African	56.0 (17.75)	**26** (<1)	22 (84.6)	19 (73.1)	4 (15.4)	8 (30.8)	16 (61.5)	10 (38.5)
Any other ethnic group	56.0 (24.0)	**111** (4.2)	83 (74.7)	76 (68.5)	28 (25.2)	31 (27.9)	95 (85.6)	16 (14.4)
Chinese	54.5 (32.5)	**16** (<1)	12 (75.0)	12 (75.0)	4 (25.0)	6 (37.5)	13 (81.3)	3 (18.8)
Bangladeshi	45.0 (38.0)	**11** (<1)	6 (54.5)	6 (54.5)	5 (45.5)	2 (18.2)	9 (81.8)	2 (18.2)
Mixed	67.5 (32.0)	**22** (<1)	15 (68.2)	11 (50.0)	7 (31.8)	2 (9.1)	14 (63.6)	8 (36.4)
Unspecified	57.0 (28.0)	**19** (<1)	15 (78.9)	12 (63.2)	4 (21.1)	3 (15.8)	15 (78.9)	4 (21.1)
ICU admission	60.00 (18.00)	**310** (11.7)	272 (87.7)	249 (80.3)	38 (12.3)			
Discharge	80.00 (17.00)	**1771** (66.9)	1037 (58.6)	797 (45.0)	734 (41.4)			
Mortality	72.0 (28.0)	**875** (33.1)	630 (72.0)	510 (58.3)	245 (28.0)			

All COVID-19 positive patients N (% of column total).

ICU, intensive care unit.

#### Comorbidities

Comorbidities including obesity, hypertension, ischaemic heart disease (IHD), heart failure, peripheral vascular disease, chronic obstructive pulmonary disease, type 2 diabetes mellitus (T2DM), liver cirrhosis and chronic kidney disease (CKD) were associated with increased mortality (online supplemental 5). Comorbidities by ethnic group are shown in online supplemental 6. CCI scores among each ethnic minority group were higher than White controls matched by age, sex and deprivation subdomain (online supplemental 7). The average number of comorbidities among African, Pakistani and Caribbean patients was higher than age-matched and sex-matched White controls. Ethnic minorities had higher average Body Mass Index (BMIs) than White patients, with the exception of Indian and Bangladeshi subgroups.

#### Deprivation: household overcrowding, adult skills, housing quality and air pollution

The proportion of patients admitted to hospital from the most deprived quintile was as follows: wider BHS (59.0%), adult skills (43.6%), indoor LE (42.3%) and outdoor LE (56.5%) (online supplemental 8). ICU admissions by deprivation subdomain are depicted in online supplemental 9.

The proportions of ethnic minorities versus White patients hospitalised from the most deprived quintile by deprivation type was as follows: wider BHS (81.7% vs 50.2%), adult skills (65.8% vs 35.1%), indoor LE (54.6% vs 37.5%) and outdoor LE (81.5% vs 46.9%). A breakdown by ethnic minority subgroup is available in online supplemental 10. Ethnic minorities were more likely than White patients to be admitted from the most deprived quintile of the aforementioned deprivation forms, present with multilobar pneumonia (OR 2.465 (95% 2.057 to 2.945); p<0.001) and require ICU admission (OR 2.823 (95% CI 2.219 to 3.611); p<0.001) (online supplemental file 1).

### Admission from highest deprivation subdomain increases risk of presentation with multilobar pneumonia

**Figure 1:**
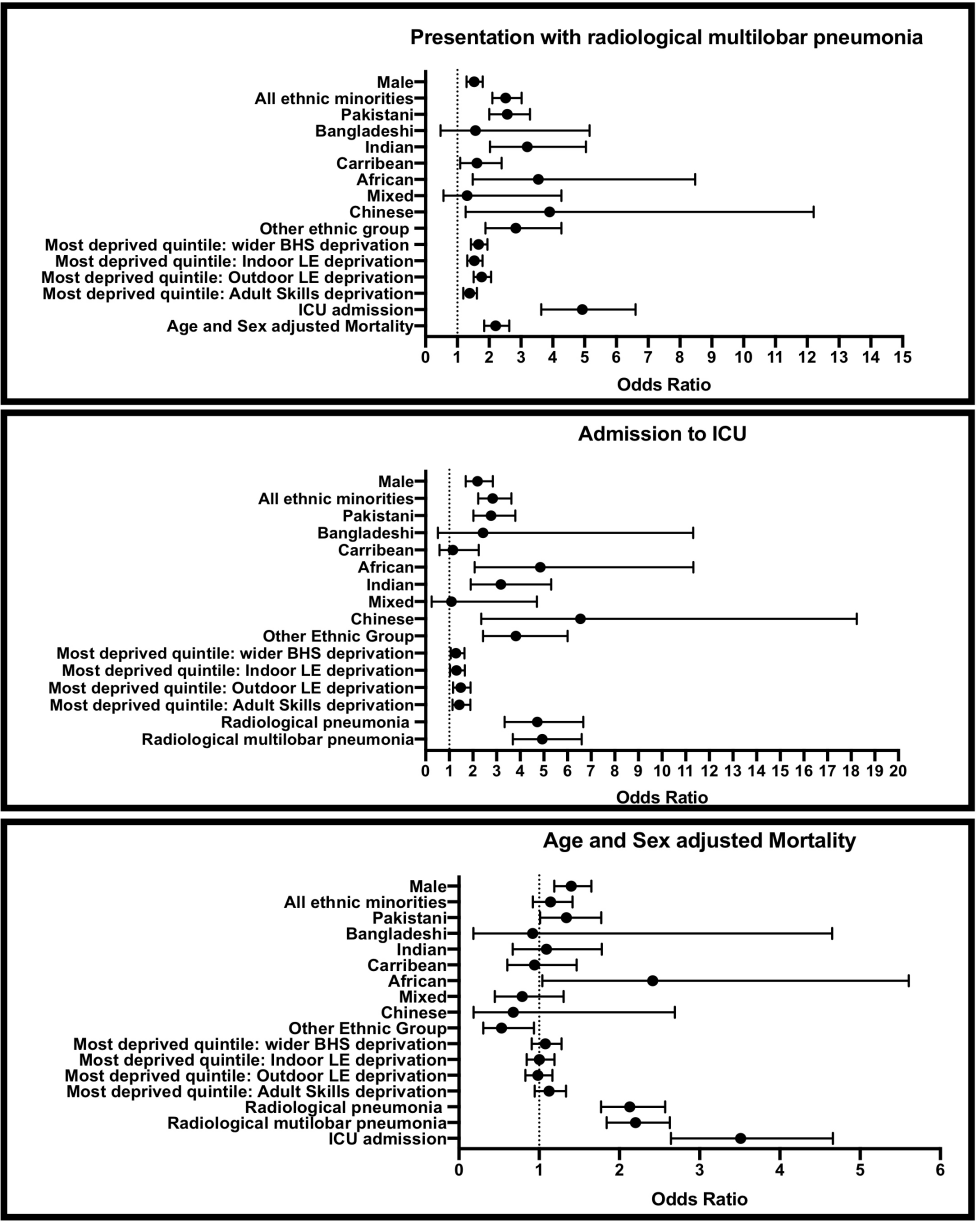
ORs of hospitalised patients with COVID-19 presenting with multilobar pneumonia, requiring ICU admission and mortality (age and sex adjusted). (A) ORs of presentation with multilobar pneumoniaby: gender, ethnicity (all ethnic minorities, Pakistani, Bangladeshi, Indian, Caribbean, African, mixed, Chinese, other ethnic group vs Caucasian), admission from most deprived quintile (wider BHS, indoor LE, outdoor LE, adult skills) versus admission from all other respective deprivation areas, admission to ICU versus not admitted to ICU and mortality (age and sex adjusted) versus discharge. (B) ORs of ICU admission by: gender, ethnicity (all ethnic minorities, Pakistani, Bangladeshi, Indian, Caribbean, African, mixed, Chinese, other ethnic group vs Caucasian), admission from the most deprived quintile (wider BHS, indoor LE, outdoor LE and adult skills) versus admission from all other respective deprivation areas and presentation with pneumonia (radiological pneumonia vs radiological multilobar pneumonia) versus presentation without pneumonia; (C) ORs of age-adjusted and sex-adjusted mortality by: gender, ethnicity (all ethnic minorities, Pakistani, Bangladeshi, Indian, Caribbean, African, mixed, Chinese, other ethnic group vs Caucasian), admission from the most deprived quintile (wider BHS, indoor LE, outdoor LE and adult skills) versus admission from all other respective deprivation areas, presentation with pneumonia (radiological pneumonia and radiological multilobar pneumonia) versus presentation without pneumonia and ICU admission versus not admittedto ICU. BHS, barriers to housing and services; LE, living environment.

Patients were more likely to present with radiological multilobar pneumonia if domiciled from the most deprived quintile: wider BHS (OR 1.66 (95% CI 1.42 to 1.95); p=0.049), indoor LE (OR 1.54 (95% CI 1.31 to 1.79); p<0.0001), outdoor LE (OR 1.76 (95% CI 1.51 to 2.06); p<0.001) and adult skills (OR 1.42 (95% CI 1.14 to 1.83); p=0.003) compared with patients admitted from all other respective quintiles (figure 1a). Patients presenting with multilobar pneumonia were more likely to require ICU admission (OR 4.93 (95% CI 3.68 to 6.60), p<0.000) and die (age and sex adjusted) (OR 2.20 (95% CI 1.84 to 2.63); p<0.000) (figure 1a).

### Admission from highest deprivation subdomain increases the risk of ICU admission

Patients were more likely to be admitted to ICU if admitted from the most deprived quintile (subdomains 1 and 2): wider BHS (OR 1.28 (95% CI 1.00 to 1.64); p=0.048), indoor LE (OR 1.31 (95% CI 1.03 to 1.66); p=0.028), outdoor LE (OR 1.49 (95% CI 1.16 to 1.90); p=0.002) and adult skills (OR 1.44 (95% CI 1.14 to 1.83); p=0.002) compared with patients admitted from all other respective quintiles (figure 1B). Age-adjusted and sex-adjusted mortality was higher among patients admitted to ICU (OR 3.51 (95% CI 2.64 to 4.66); p<0.000) (figure 1B).

### Ethnic minorities: IMD subdomains, presentation and ICU admission

#### Indian

**Figure 2:**
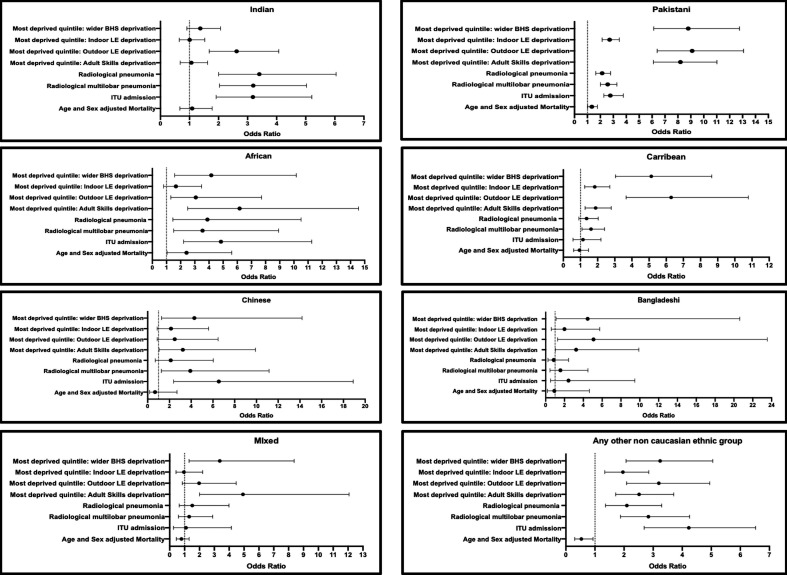
ORs of hospitalised COVID-19 positive patients of (A) Pakistani, (B) Indian, (C) Bangladeshi, (D) African, (E) Caribbean, (F) Chinese, (G) mixed and (H) any other ethnicity by: admission from the mostdeprived quintile (wider BHS, indoor LE, outdoor LE, adult Skills), ITU admission and mortality (age and sex adjusted). BHS, barriers to housing and services; LE, living environment.

Indian patients were more likely than White patients to be admitted from the most deprived quintile: outdoor LE deprivation (OR 2.62 (95% CI 1.68 to 4.07); p<0.001), present with multilobar pneumonia (OR 3.20 (95% CI 2.03 to 5.03); p<0.001) and require ICU admission (OR 3.18 (95% CI 1.91 to 5.31); p<0.001) (figure 2A).

#### Pakistani

Pakistani patients were more likely than White patients to be admitted from the most deprived quintile: wider BHS (OR 8.80 (95% CI 6.13 to 12.76); p<0.001), outdoor LE (OR 9.10 (95% CI 6.39 to 13.08); p<0.001), indoor LE (OR 2.71 (95% CI 2.14 to 3.46); p<0.001), adult skills (OR 8.20 (95% CI 6.10 to 11.02); p<0.001), present with multilobar pneumonia (OR 2.57 (95% 2.01 to 3.28); p<0.001) and require ICU admission (OR 2.77 (95% CI 2.02 to 3.79); p<0.000) (figure 2B).

#### African

Africans were more likely than White patients to be admitted from the most deprived quintile: wider BHS (OR 4.16 (95% CI 1.58 to 10.17); p=0.002), outdoor LE (OR 3.07 (95% CI 1.31 to 7.72); p=0.009), adult skills (OR 6.16 (95% CI 2.50 to 14.57); p<0.001), present with multilobar pneumonia (OR 3.55 (1.51–8.92); p=0.004) and require ICU admission (OR 4.85 (95% CI 2.08 to 11.32); p<0.000) (figure 2C).

#### Caribbean

Caribbean patients were more likely than White patients to be admitted from the most deprived quintile: wider BHS (OR 5.13 (95% CI 3.04 to 8.65); p<0.001), indoor LE (OR 1.83 (95% CI 1.25 to 2.71); p=0.003), outdoor LE (OR 6.29 (95% CI 3.66 to 11.05); p<0.001), adult skills (OR 1.88 (95% CI 1.28 to 2.78); p=0.002) and present with multilobar pneumonia (OR 1.61 (95% CI 1.09 to 2.40); p=0.020) (figure 2D). Caribbean patients were not more likely to require ICU admission (p>0.05).

#### Chinese

Chinese patients were more likely than White patients to be admitted from the most deprived quintile: wider BHS (OR 4.29 (95% CI 1.27 to 14.20); p=0.021), present with multilobar pneumonia (OR 3.92 (95% CI 1.26 to 11.16); p=0.020) and require ICU admission (OR 6.54 (95% CI 2.35 to 18.24); p<0.000) (figure 2E).

#### Bangladeshi

Bangladeshi patients were more likely than White patients to be admitted from the most deprived quintile: wider BHS (OR 4.46 (95% CI 1.11 to 20.63); p=0.037), outdoor LE (OR 5.09 (95% CI 1.27 to 23.53; p<0.001) and adult skills (OR 3.24 (95% CI 1.04 to 9.91); p=0.048) although they were not more likely to present with multilobar pneumonia or require ICU admission (figure 2F).

#### Mixed

Mixed ethnicity patients were more likely than White patients to be admitted from the most deprived quintile: wider BHS (OR 3.37 (95% CI 1.30 to 8.37); p=0.016) and adult skills (OR 4.93 (95% CI 2.01 to 12.07); p=0.001) although they were not more likely to present with multilobar pneumonia or require ICU admission (figure 2G).

#### Any other non-White ethnic group

Patients of any other non-White ethnicity were more likely than White patients to be admitted from the most deprived quintile: wider BHS (OR 3.24 (95% CI 2.07 to 5.06); p<0.001), indoor LE (OR 1.96 (95% CI 1.34 to 2.85); p<0.001), outdoor LE (OR 3.20 (95% CI 2.08 to 4.95); p<0.001), adult skills (OR 2.52 (95% CI 1.71 to 3.71); p<0.001), present with multilobar pneumonia (OR 2.84 (95% CI 1.88 to 4.25); p<0.001) and require ICU admission (OR 3.82 (95% CI 2.43 to 6.01); p<0.000) (**figure 2h**).

### Risk factors for mortality

Multivariate analysis including variables shown in online supplemental 3 identified seven variables that were independently associated with mortality: age, sex, obesity, cirrhosis, Ischaemic Heart Disease (IHD), CCI score and presentation with multilobar pneumonia.

### Clinical risk stratification tools

**Figure 3:**
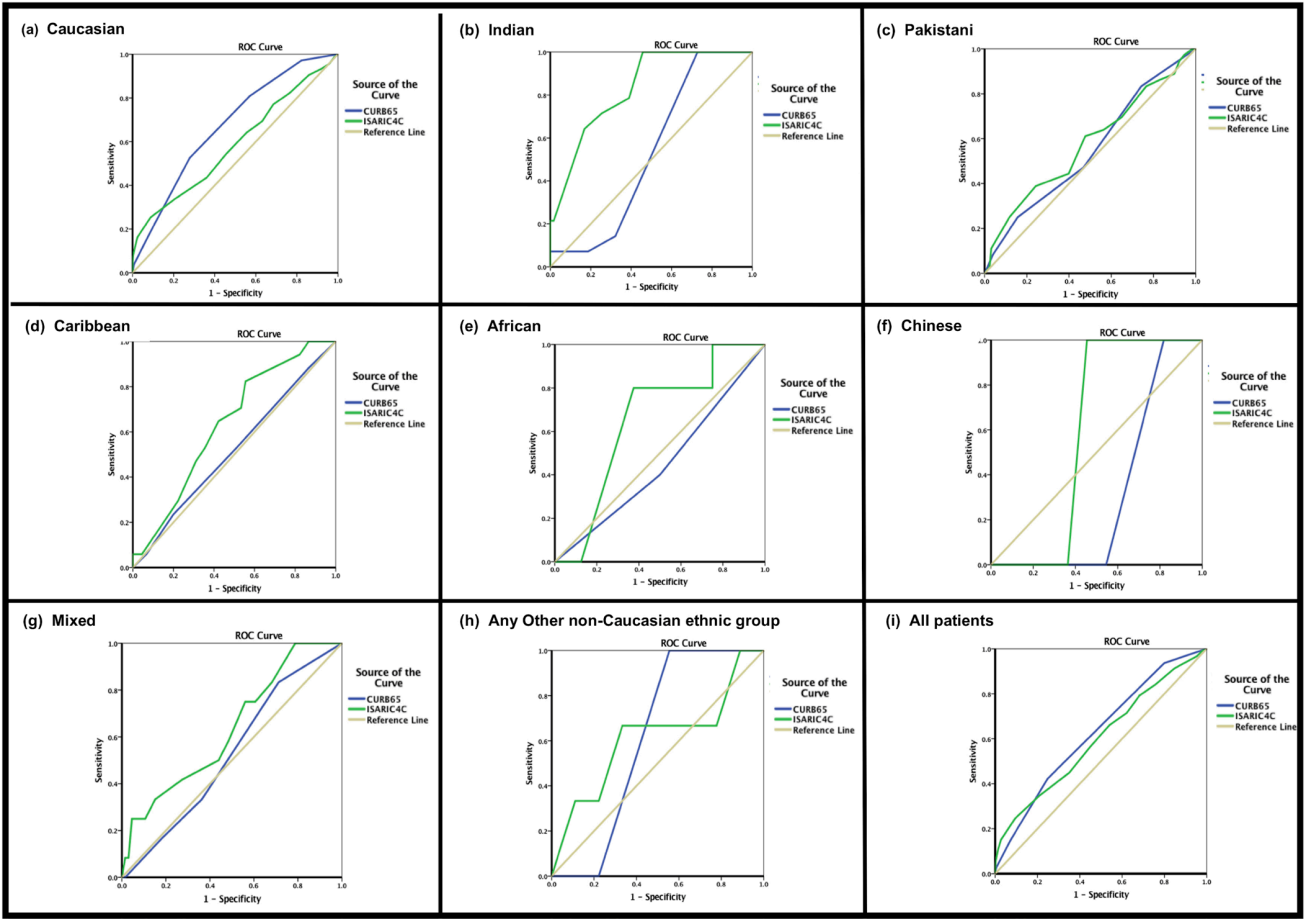
Graphs showing receiver operating characteristics curve for the CURB65 and ISARIC 4Cscores by ethnicity: (A) Caucasian, (B) Indian, (C) Pakistani, (D) Caribbean, (E) African, (F) Chinese, (G) mixed, (H) any other ethnic group and (I) all patients.

AUROC was used to test the performance of the CURB65 and ISARIC 4C scores in predicting in-hospital mortality by ethnic group. Highest AUROC curves were achieved by the ISARIC4C score for the prediction of in-hospital mortality among Indian patients (OR 0.83; 95% CI 0.73 to 0.93). Area under the curve (AUC) did not exceed 0.7 for CURB65 or ISARIC4C among any of the other ethnic groups (figure 3 and online supplemental 12).

Ethnic minorities with pneumonia and low CURB65 scores (0–1) had higher mortality than White patients (OR 22.6% vs 9.4%; p<0.001); Africans were at highest risk 38.5% (OR 6.05 (95% CI 2.13 to 18.89); p=0.006), followed by Caribbean 26.7% (OR 3.52 (95% CI 1.53 to 8.45); p=0.008), Indian 23.1% (OR 2.90 (95% CI 1.43 to 6.07); p=0.007) and Pakistani 21.2% (OR 2.56 (95% CI 1.42 to 4.66); p=0.004). Table 2 disaggregates CURB65 scores by ethnic group.

**Table 2:** CURB65 scores among patients with COVID-19 presenting with pneumonia by ethnic group

	Number of patients presenting with pneumonia	Number of patients with CURB65 score data	CURB65 score 0–1			CURB65 score 2			CURB65 scores 3–5		
			Total no N (% of total)	Died N (% of patients with CURB65 0–1)	Discharged N (% of patients with CURB65 0–1)	Total no N (% of total)	Died N (% of patients with CURB65 2)	Discharged N (% of patients with CURB65 2)	Total no N (% of total)	Died N (% of patients with CURB65 3–5)	Discharged N (% of patients with CURB65 3–5)
White	1123	1110	427 (38.5)	40 (9.4)	387 (90.6)	322 (29.0)	59 (18.3)	263 (81.7)	361 (32.5)	110 (30.5)	251 (69.5)
Ethnic minorities	529	419	252 (60.1)	57 (22.6)	196 (77.8)	92 (22.0)	16 (17.4)	77 (83.7)	76 (18.1)	16 (21.1)	58 (76.3)
Pakistani	245	163	85 (52.1)	18 (21.2)	68 (81.9)	46 (28.2)	8 (17.4)	39 (84.8)	32 (19.6)	9 (28.1)	21 (61.8)
Indian	77	73	52 (71.2)	12 (23.1)	40 (76.9)	9 (12.3)	1 (11.1)	8 (88.9)	12 (2.7)	1 (8.3)	11 (91.7)
Caribbean	69	62	30 (48.4)	8 (26.7)	22 (73.3)	19 (30.6)	5 (26.3)	14 (73.7)	13 (21.0)	4 (30.8)	9 (69.2)
African	22	13	13 (100)	5 (38.5)	8 (61.5)	0 (0)	0 (0)	0 (0)	0 (0)	0 (0.0)	0 (0.0)
Chinese	12	12	6 (50.0)	1 (16.7)	5 (83.3)	3 (25.0)	0 (0)	3 (100)	3 (25.0)	0 (0.0)	3 (100)
Bangladeshi	6	5	5 (100)	0 (0.0)	5 (100)	0 (0)	0 (0)	0 (0)	0 (0)	0 (0.0)	0 (0.0)
Mixed	15	12	8 (76.9)	2 (25.0)	6 (75.0)	2 (7.7)	1 (50.0)	1 (50.0)	2 (15.4)	0 (0.0)	2 (100)
Any other ethnic group	83	79	51 (64.6)	9 (17.6)	42 (82.4)	14 (17.7)	2 (14.3)	12 (85.7)	14 (17.7)	2 (14.3)	12 (85.7)
Unspecified	15	15	9 (60.0)	0 (0.0)	9 (100)	5 (33.3)	1 (20.0)	4 (80.0)	1 (6.7)	0 (0)	1 (100)
Total	1667	1544									

## Discussion

Ethnic minorities are more likely to be hospitalised with COVID-19 from areas of highest deprivation. Admission from areas of highest indoor LE deprivation, outdoor LE deprivation, wider BHS deprivation and adult skills deprivation are associated with multilobar pneumonia on presentation and ICU admission, which are mortality risk factors. Deprivation metrics are not incorporated within current clinical admission risk stratification tools for hospitalised patients with COVID-19. This may explain the higher ICU admissions among ethnic minorities reported by ICNARC and ONS data reporting higher age standardised mortality rates among patients in the most deprived IMD areas.^1 10^

Socioenvironmental risk factors have long been neglected from our frontline clinical risk stratification of acutely unwell patients including patients with COVID-19 pneumonia, despite a body of literature demonstrating the health risks. First, air pollutants are known to compromise the host’s immune response against invading pathogens in the respiratory tract.^17^ Chronic exposure to nitrogen dioxide and sulphur dioxide concentrations are associated with incidence of pneumonia,^18^ while particulate matter increases the activity of ACE 2 receptors on cell surfaces,^19^ thus enhancing COVID-19 uptake by the lungs. Second, household overcrowding and housing quality failing to meet the Decent Homes Standard has been linked to an increased risk of exposure to and spread of pathogenic species including bacteria, fungal and viral pathogens as well as an increased incidence of pneumonia.^20 21^ National UK studies have recorded associations between: (A) household overcrowding and testing positive for COVID-19^22^ and (B) household overcrowding involving a multigenerational household and increased mortality from COVID-19 amounting to a 10%–15% elevated risk among older females from South Asian background.^23^ Third, cultural variations, language barriers and adult qualification levels contribute to delayed symptom identification, reporting and/or presentation with coronavirus resulting in an increased risk of multilobar pneumonia on presentation.^24^ Minimising deprivation inequalities in air pollution, household overcrowding, housing quality and adult skills is essential to reduce the disease burden of COVID-19 community acquired pneumonia.^25 26^ Meanwhile, capturing these hidden socioenvironmental risk factors within our admission clinical risk stratification tools is essential for ensuring that admission risk tools reflect risk factors to which patients from a range of demographic backgrounds are exposed with resultant triage to the appropriate level of care.

Furthermore, more needs to be done to ensure that admission clinical risk tools account for factors to which ethnic minorities are predominantly predisposed. Ethnic minorities exhibit younger epidemiological age structures that result in underscoring using the 232 diagnostic or prognostic clinical risk stratification tools identified in a relevant systematic review.^3^ Moreover, despite presenting with younger age structures, ethnic minorities present with higher CCI scores and a higher incidence of obesity yet neither factor is accounted for in commonly used COVID-19 admission clinical risk stratification tools. Clusters of disease are known to increase mortality,^27^ and affect ethnic groups differently,^28^ yet current COVID-19 admission clinical risk tools do not account for clusters of disease or CCI scores despite warning from the UK’s Chief Medical Officer regarding rising multimorbidity and the resultant challenges for acute and long-term care provision.^29^ Hospitalised COVID-19 patients with underlying obesity, hypertension, IHD, heart failure, Chronic Kidney Disease (CKD), Peripheral Vascular Disease (PVD), Type 2 Diabetes Mellitus (T2DM) and cirrhosis are at increased risk of mortality.

The oversight of scoring biological, demographic and socioenvironmental risk factors to which ethnic minorities are predominantly predisposed results in potential underscoring and triage to an inappropriate level of care, while clinicians are left falsely reassured regarding the severity of presentation and risk of deterioration.

It is perhaps therefore not surprising that the AUROC analyses demonstrated generally poor performance of the CURB65 and ISARIC 4C admission risk stratification tools among individual ethnic groups hospitalised with COVID-19. The only exception was the optimum performance of the ISARIC 4C tool in predicting mortality among the Indian cohort, which was domiciled from areas of relatively lower deprivation profiles compared with other ethnic minorities. Ethnic minorities presenting with pneumonia and low CURB65 scores (0–1) have higher mortality than White patients; Africans are at highest risk, followed by Caribbean, Indian and Pakistani. The findings in this study are consistent with those of a recent study of COVID-19 pneumonia patients (n=279), which found that, as a largely physiological assessment, CURB65 is an unreliable mortality risk tool in COVID-19 pneumonia.^30^ Generally, ISARIC4C exhibits better performance among hospitalised ethnic minorities than CURB65, which is likely to be in part due to its inclusion of some risk factors to which ethnic minorities are predisposed: scoring >2 comorbidities, CRP and oxygen saturations. The latter two assessment metrics are typical of presentation with pneumonia. 31 32

While socioenvironmental deprivation metrics are not included within current admission risk tools, the community-based QCOVID tool for predicting hospital admission incorporates the Townsend deprivation score, which contains indicators for unemployment, household overcrowding, and car and home ownership.^33^ However, a limitation of the Townsend score is the absence of air pollution data, housing quality data or adult skills data that are risk factors for presentation with multilobar pneumonia and ICU admission. Yet, it is true to say that no assumptions can be made about the exposure of a given individual to constituent risk factors within the Townsend score, IMD, its domains and subdomains, as these rely on Census data by geographical area or postcode. This paper uses the most granular level of IMD deprivation metrics available, namely, IMD subdomains. While the IMD considers multiple national sets of data to come up with an overall rank for deprivation factors and is the official measure of relative deprivation for small areas in England, a limitation of the IMD is that the outdoor LE subdomain includes indicators for both air pollution and road traffic accidents. We believe that consideration should be given to separating these two indicators especially in light of the Ella Kissi Debra case and the Preventing Future Deaths Report.^34^

An important message from this study is that individual ethnic minorities exhibit distinct risk factor profiles. Although this study includes hospitalised patients with COVID-19 within four hospitals across the West Midlands constituting one of the UK’s largest National Health Service Trusts, one of the challenges of analysing ethnic minority group data relates to small groups and wide CIs that adds a level of uncertainty introducing a need for interpreting small cohorts with caution.

A surprising finding is that Caribbean patients did not appear to be at increased risk of mortality despite presenting 17 years older than African patients. This was despite both groups exhibiting a similarly high multimorbidity burden and being more likely than White patients to be admitted from areas of highest wider BHS deprivation, outdoor LE deprivation and adult skills deprivation. Nevertheless, several hypotheses have been put forward to explain the increased mortality among Africans including the high prevalence of glucose-6-phosphate dehydrogenase deficiency which, it has been suggested, may increase viral replication and susceptibility to viral infections by inducing oxidative stress; antioxidants have been found to be protective against viral infection.^35^ Further studies are needed to explore genetic, immunological and metabolic differences between African and Caribbean groups.

## Conclusion

Ethnic minorities exhibit younger age structures, higher multimorbidity and disproportionate exposure to unscored risk factors including obesity and deprivation resulting in potential triage to an inappropriate level of care with clinicians left falsely reassured regarding the severity of presentation and risk of deterioration. Household overcrowding deprivation, air pollution deprivation, housing quality deprivation and adult skills deprivation are associated with multilobar pneumonia on presentation and ICU admission. Risk tools need to reflect risk factors predominantly affecting ethnic minorities.

Consideration of multiethnic age structures, sex, body mass index, CCI score, chest X-ray imaging and deprivation subdomains on admission supports clinicians in stratifying high-risk patients. COVID-19 admission clinical risk stratification tools need to be developed to account for risk factors to which ethnic minorities are predominantly exposed. This will enable the early identification of patients at risk of deterioration and ensure triage to an appropriate level of care.

Future studies need to relate these findings with populations from other urban rural areas with this level of granularity to inform national strategic planning on risk stratification and minimising health inequalities.

## Data Availability

All data relevant to the study are included in the article or uploaded as supplementary information.
