# Supplementary material for: COVID-19 admission risk tools should include multiethnic age structures, multimorbidity and deprivation metrics for air pollution, household overcrowding, housing quality and adult skills
Source: BMJ Open Respir Res. 2021 Aug 9;8(1):e000951. doi: 10.1136/bmjresp-2021-000951 (PMC8354812; doi:10.1136/bmjresp-2021-000951)
Supplement: Supplementary data [file bmjresp-2021-000951supp001.pdf]

Online supplement 1

Patient management

Patients were admitted and treated initially according to British Thoracic Society (BTS) guidelines for COVID19 community acquired pneumonia with antibiotics, fluids and controlled oxygen where appropriate. Trust infection prevention measures were followed. No experimental agents were administered to these patients outside of clinical trials. A limited number of patients were enrolled in the UK RECOVERY trial and a trial of inhaled IFN-beta1a in COVID19 disease. No patients received ward-based continuous positive airway pressure non-invasive ventilation. Ward based bi-level non-invasive ventilation was only used if patients with pre-existing causes for chronic type-two respiratory failure were admitted with acute respiratory acidosis, with no evidence of infiltrates on their chest x-ray. At the beginning of the pandemic, the trust introduced a rapid review Chest X-ray reporting service staffed by Consultant radiologists to ensure Chest X-rays were reported within 12 hours of being undertaken. All suspected COVID19 infected patients had a decision about escalation to critical care and discussion in relation to resuscitation status at their first review after admission (typically in less than 4 hours due to the introduction of resident consultants during the pandemic). Patients who were for critical care escalation were reviewed by the critical care assessment team if they had an altered GCS, persistently low systolic blood pressure (<90mmgHg), respiratory acidosis (pH<7.2) or were unable to maintain their target saturations or had a respiratory rate >30 breaths per minute despite receiving a fractional inspired oxygen (FiO_2) of ≥ 0.5. If deemed appropriate, patients were intubated and transferred to critical care subsequently. All patients were prescribed their regular medications for existing medical conditions whilst in hospital unless a medication was contraindicated for clinical reasons in which case it was paused temporarily until safe to resume. All patients received 40 mg subcutaneous enoxaparin as venous thromboembolic disease prophylaxis daily, unless it was contraindicated, as per our hospital policy.

Online supplement 2

Authors	Score name	Country of derivation	Development population	Pre-existing or COVID specific	Model outcome	Predictors	Original modelling approach	How are predictors combined?
Lim et al.	CURB65	UK, Netherlands, New Zealand	Patients with community acquired pneumonia	Pre-existing community acquired pneumonia	30 day mortality	New onset confusion, urea (>7mmol/L), respiratory rate (≥30 breaths/minute), blood pressure (<90mmHg systolic or ≤60mmHg diastolic) and age (≥65 years).	Logistic regression	Points based score
Gupta et al.	ISARIC4C mortality score	UK, France, Netherlands, Italy, Pakistan, Turkey, Canada	Patients admitted with COVID19	COVID specific	In hospital mortality	Age, Gender, Number of comorbidities, Respiratory Rate, Oxygen Saturations on room air, GCS, Urea, CRP	Logistic regression	Points based score

Characteristics of studies describing CURB65 and ISARIC4C mortality models

Online Supplement 3

	Odds Ratio	99% CI Lower bound	99% CI Upper bound	p-value
**Univariate**				
Age	1.04	1.03	1.04	<0.000
Male sex	1.40	1.19	1.40	<0.000
Obesity (BMI ≥ 30)	3.32	2.77	3.96	<0.001
Hypertension	1.23	1.04	1.45	0.018
Ischaemic Heart Disease	1.34	1.08	1.66	<0.009
Heart Failure	1.38	1.03	1.84	<0.032
Peripheral Vascular Disease	1.81	1.10	2.95	<0.022
COPD	1.34	1.03	1.73	<0.034
Type 2 Diabetes Mellitus	1.21	1.01	1.45	<0.041
Cirrhosis	4.40	1.79	10.82	<0.0009
Chronic Kidney Disease	1.44	1.14	1.81	<0.002
Charlson Comorbidity (CCI) Score	1.19	1.16	1.23	<0.000
Multilobar pneumonia	2.13	1.77	2.57	<0.000
Index of Multiple Deprivation	0.88	0.75	1.04	0.126
Wider BHS deprivation	0.92	0.78	1.08	0.305
Outdoor LE deprivation	0.85	0.72	0.99	0.042
Indoor LE deprivation	0.92	0.78	1.08	0.288
Adult Skills deprivation	0.91	0.77	1.07	0.268
Ethnic minorities	0.69	0.57	0.84	<0.000
Pakistani	1.34	1.01	1.77	<0.041
African	2.42	1.04	5.61	<0.040
Carribean	0.94	0.60	1.47	0.787
Indian	0.92	0.56	1.50	0.726
Bangladeshi	0.92	0.18	4.65	0.917
Chinese	0.68	0.18	2.51	0.559
Mixed	1.72	0.68	4.34	0.255
Any other ethnic group	0.53	0.30	0.93	0.028
**Multivariate**				
Age	1.05	1.04	1.06	<0.000
Male sex	1.50	1.25	1.81	<0.000
Charlson Comorbidity (CCI) Score	1.11	1.06	1.16	<0.000
Obesity (BMI ≥ 30)	3.60	2.95	4.38	<0.000
Ischaemic Heart Disease	0.78	0.60	0.99	0.047
Cirrhosis	9.72	3.47	27.17	<0.000
Multilobar pneumonia	1.89	1.56	2.28	<0.000

Univariate and multivariate analyses to predict mortality

Online Supplement 4

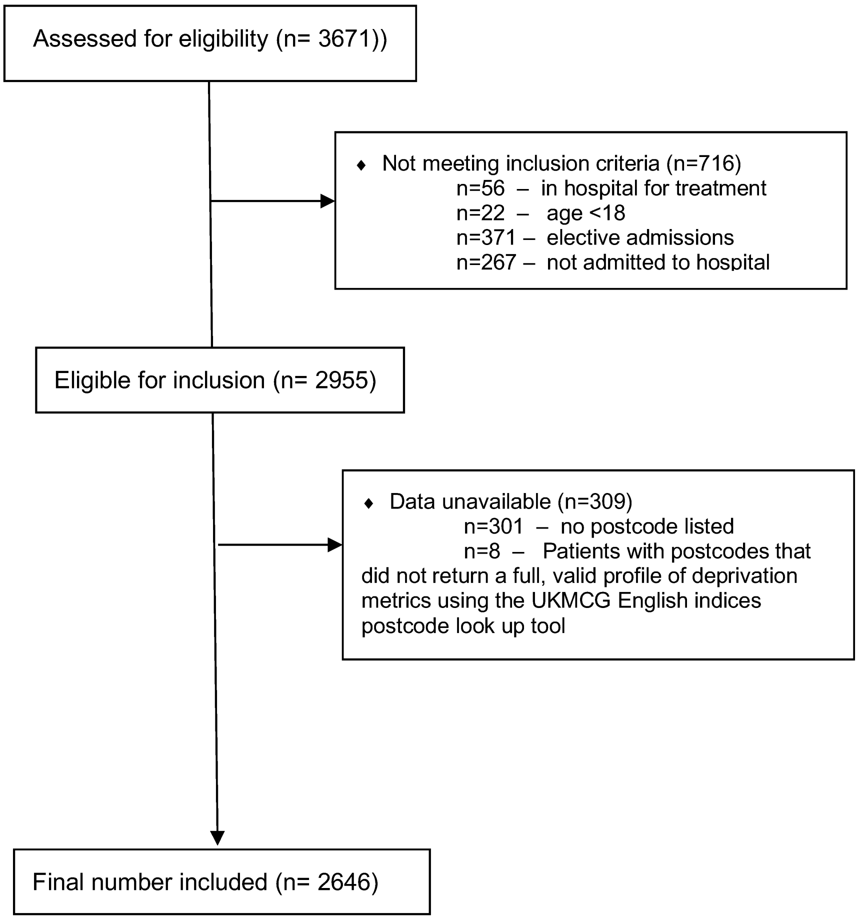

A CONSORT diagram showing participants assessed for eligibility, the inclusion criteria and the final number of participants included. 3671 consecutive patients were assessed for eligibility for inclusion into this study. 716 patients were excluded on account of having not met the inclusion criteria due to: ongoing hospitalisation on 1st September 2020(n=55), age <18 (n=22), attending hospital as an elective admission(n=371) or attending hospital without admission(n=267). Patients eligible for inclusion in this study(n=2955) were reviewed; patients without listed postcodes(n=301) or postcodes not returning deprivation metrics(n=8) could not be included in the analysed group(n=2646).

Online Supplement 5

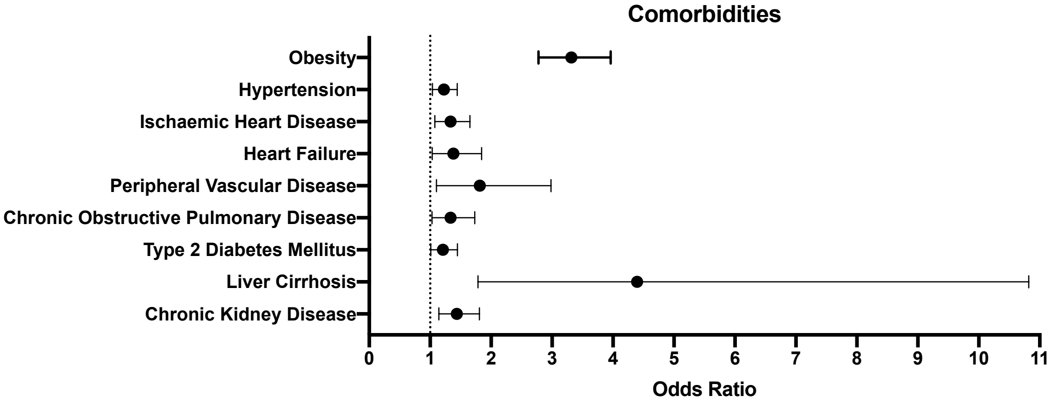

Odds ratios of mortality among COVID-19 patients by underlying obesity (BMI>30), hypertension, ischaemic heart disease, heart failure, peripheral vascular disease, COPD, type 2 diabetes mellitus, liver cirrhosis and chronic kidney disease

Online Supplement 6

	All COVID-19-positive patients	White	Ethnic minorities	Pakistani	Indian	Caribbean	African	Chinese	Bangladeshi	Mixed	Any other ethnic group
	2646	1917 (72.4)	710 (26.8)	326 (12.3)	93 (3.5)	105 (4.0)	26 (<1)	16 (<1)	11 (<1)	22 (<1)	111 (4.2)
**Cardiovascular (n, % of column)**											
HTN	1030 (38.9)	736 (38.4)	266 (37.4)	130 (39.9)	43 (46.2)	53 (50.5)	9 (34.6)	5 (31.3)	2 (18.2)	6 (27.3)	40 (36.0)
IHD	433 (16.4)	317 (16.5)	113 (15.9)	53 (16.3)	14 (15.1)	15 (14.3)	5 (19.2)	0 (0)	0 (0)	20 (90.9)	6 (5.4)
Hypercholesterolaemia	206 (7.8)	145 (7.6)	56 (7.9)	33 (10.1)	4 (4.3)	6 (5.7)	3 (11.5)	2 (12.5)	1 (9.1)	6 (27.3)	1 (0.9)
CCF	208	166 (8.7)	42 (5.9)	24 (7.4)	5 (5.4)	6 (5.7)	0 (0)	0 (0)	0 (0)	6 (27.3)	1 (0.9)
Peripheral Vascular disease	64 (2.4)	56 (2.9)	8 (1.1)	2 (0.6)	1 (1.1)	5 (4.8)	0 (0)	0 (0)	0 (0)	0 (0)	0 (0)
Stroke	215 (8.1)	170 (8.9)	44 (6.2)	12 (3.7)	3 (3.2)	15 (14.3)	2 (7.7)	1 (6.3)	1 (9.1)	7 (31.8)	3 (2.7)
**Respiratory (n, % of column)**											
Asthma	260 (9.8)	194 (10.1)	66 (9.3)	31 (9.5)	8 (8.6)	9 (8.6)	2 (7.7)	1 (6.3)	1 (9.1)	10 (45.5)	3 (2.7)
COPD	269 (10.2)	202 (10.5)	67 (9.4)	28 (8.6)	9 (9.7)	8 (7.6)	3 (11.5)	2 (12.5)	0 (0)	12 (54.5)	3 (2.7)
ILD	53 (2.0)	42 (2.2)	11 (1.5)	4 (1.2)	1 (1.1)	2 (1.9)	1 (3.8)	1 (6.3)	0 (0)	2 (9.1)	0 (0)
OSA	51 (1.2)	38 (2.0)	13 (1.8)	5 (1.5)	2 (2.2)	2 (1.9)	0 (0)	1 (6.3)	0 (0)	3 (13.6)	0 (0)
Bronchiectasis	32 (1.2)	27 (1.4)	5 (0.7)	3 (0.9)	0 (0)	0 (0)	1 (3.8)	0 (0)	0 (0)	0 (0)	0 (0)
**Renal (n, % of column)**											
CKD	355 (13.4)	262 (13.7)	93 (13.1)	39 (12.0)	14 (15.1)	11 (10.5)	3 (11.5)	1 (6.3)	2 (18.2)	15 (68.2)	6 (5.4)
**Endocrinology (n, % of column)**											
T1DM	26 (1.0)	23 (1.2)	3 (0.4)	2 (0.6)	0 (0)	0 (0)	0 (0)	1 (6.3)	0 (0)	0 (0)	0 (0)
T2DM	713 (26.9)	517 (27.0)	196 (27.6)	81 (24.8)	29 (31.2)	26 (24.8)	5 (19.2)	2 (12.5)	2 (18.2)	6 (27.3)	8 (7.2)
Vitamin D < 20	56 (2.1)	38 (2.0)	18 (2.5)	7 (2.1)	4 (4.3)	3 (2.9)	2 (7.7)	0 (0)	0 (0)	2 (9.1)	0 (0)
BMI>30	742 (28.0)	481 (25.1)	261 (36.8)	136 (41.7)	22 (23.7)	34 (32.4)	10 (38.5)	5 (31.3)	2 (18.2)	8 (36.4)	35 (31.5)
**Hepatobiliary (n, % of column)**											
Hepatitis	14 (0.5)	4 (0.2)	10 (1.4)	4 (1.2)	4 (4.3)	1 (1.0)	2 (2.7)	1 (6.3)	0 (0)	1 (4.5)	1 (0.9)
Cirrhosis	22 (0.8)	21 (1.1)	1 (0.1)	0 (0)	0 (0)	0 (0)	0 (0)	0 (0)	0 (0)	0 (0)	1 (0.9)
Peptic Ulcer Disease	35 (1.3)	30 (1.6)	5 (0.7)	2 (0.6)	1 (1.1)	0 (0)	0 (0)	0 (0)	0 (0)	1 (4.5)	0 (0)
Variceal GI bleed	12 (0.5)	10 (0.5)	2 (0.3)	0 (0)	0 (0)	1 (1.0)	0 (0)	0 (0)	0 (0)	1 (4.5)	0 (0)
**Rheumatology (n, % of column)**											
Connective Tissue Disease	204 (7.7)	166 (8.7)	38 (5.4)	15 (4.6)	5 (5.4)	8 (7.6)	3 (11.5)	3 (18.8)	0 (0)	4 (18.2)	0 (0)
**Multimorbidity (n,% of column)**											
>1 comorbidity	2042 (81.4)	1555 (84.7)	472 (66.5)	209 (69.4)	66 (75.0)	87 (85.3)	19 (76.0)	12 (80.0)	5 (45.5)	14 (77.8)	74 (66.7)
4 or more comorbidities	791 (31.6)	641 (31.6)	147 (20.7)	68 (22.6)	16 (18.2)	31 (30.4)	6 (24.0)	1 (6.7)	0 (0)	3 (16.7)	25 (22.5)

A table representing underlying comorbidities and multimorbidity among hospitalised COVID-19 positive patients by ethnic subgroup: disaggregating ethnic minorities

Online Supplement 7

Case control matching by		n	Pakistani	Control	n	African	Control	n	Caribbean	Control	n	Indian	Control	n	Chinese	Control	n	Bangladeshi	Control	n	Mixed	Control	n	Any other ethnic group	Control
**A) Age, Gender**	Charlson Comorbidity Score **Median (IQR)**	295	5(5)	4(4)	26	4(6)	2.5(4)	103	6(5)	5(5)	89	5(4)	4(4)	15	4(4)	3(5)	8	2(5)	2(5)	21	5(5)	5(5)	106	3(4.25)	2(4.25)
	No. of Comorbidities **Median (IQR)**	295	4(3)	3(4)	26	4(3)	3(3)	103	4(3)	3(3.75)	89	3(2)	3(3.75)	15	2(1.75)	2(2)	8	1(3)	2(3)	21	3(2.5)	3(3)	106	2(3)	2(3)
**B) Age, Gender and Outdoor LE deprivation**	Charlson Comorbidity Score **Median (IQR)**	265	5(4)	4(4)	25	5(3)	4(6)	101	6(5)	5(4)	82	5(3)	4(5)	15	4(4.5)	3.5(7)	9	4.5(7.25)	2(4)	20	5.5(5.25)	4.5(4.75)	93	4(4.25)	3(3)
**C) Age, Gender and Indoor LE deprivation**	Charlson Comorbidity Score **Median (IQR)**	269	5(4)	4(4)	21	5(6)	4(4)	101	6(5)	5(4.5)	85	5(4)	4(4)	15	4(4.5)	3(3.5)	7	4(6.5)	3(4)	20	5.5(5.25)	4.5(3.75)	95	4(4)	3(4)
**D) Age, Gender and Wider BHS deprivation**	Charlson Comorbidity Score **Median (IQR)**	267	5(4)	4(4)	25	4(6)	2(4)	100	6(4.75)	5(4)	85	5(3.5)	4(4.5)	15	4(4)	3(5)	9	5(7)	3(4)	20	5.5(5.25)	4.5(4.75)	95	4(4.5)	3(4)
**E) Age, Gender and Adult Skills deprivation**	Charlson Comorbidity Score **Median (IQR)**	256	5(4)	4(4)	23	5(6)	3(4)	101	6(5)	5(5)	86	5(4)	4(4)	14	4(4.5)	3.5(3.5)	9	4(6)	3.5(6)	10	4.5(3.5)	3.5(3.25)	90	3(4)	2.5(4.25)

A table representing Charlson Comorbidity Index (CCI) Scores among patients of ethnic minorities in comparison with matched controls by: a) Age and Gender, b) Age, Gender and Outdoor LE deprivation, c) Age Gender and Indoor LE deprivation, d) Age, Gender and Adult Skills deprivation.

Online Supplement 8

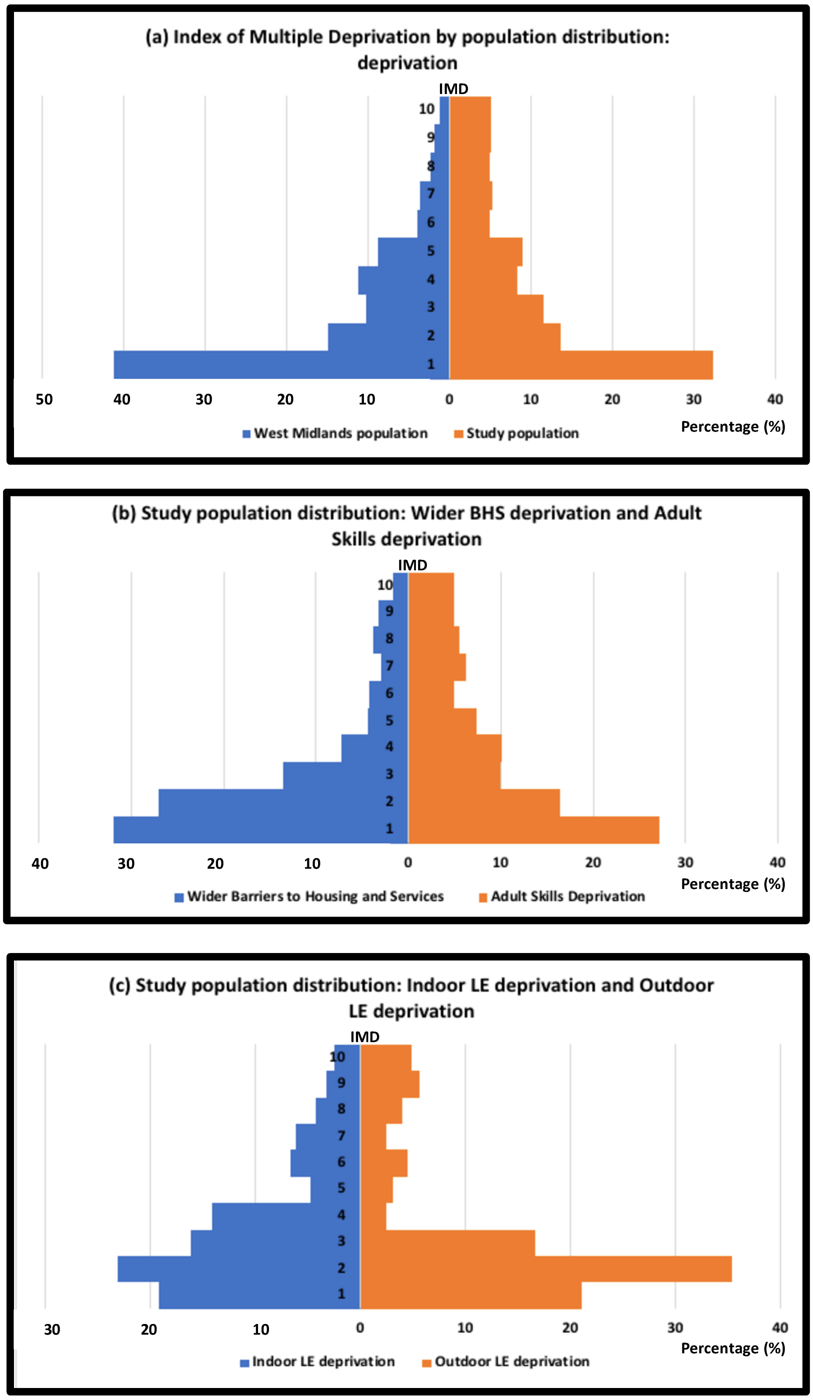

Population pyramid distributions of hospitalised COVID-19 positive patients: (a) Index of Multiple Deprivation (IMD) distribution in the West Midlands population in comparison with the study population, (b) Wider BHS and Adult skills deprivation distribution in the study population, (c) Indoor LE and Outdoor LE deprivation distribution in the study population.

Online Supplement 9

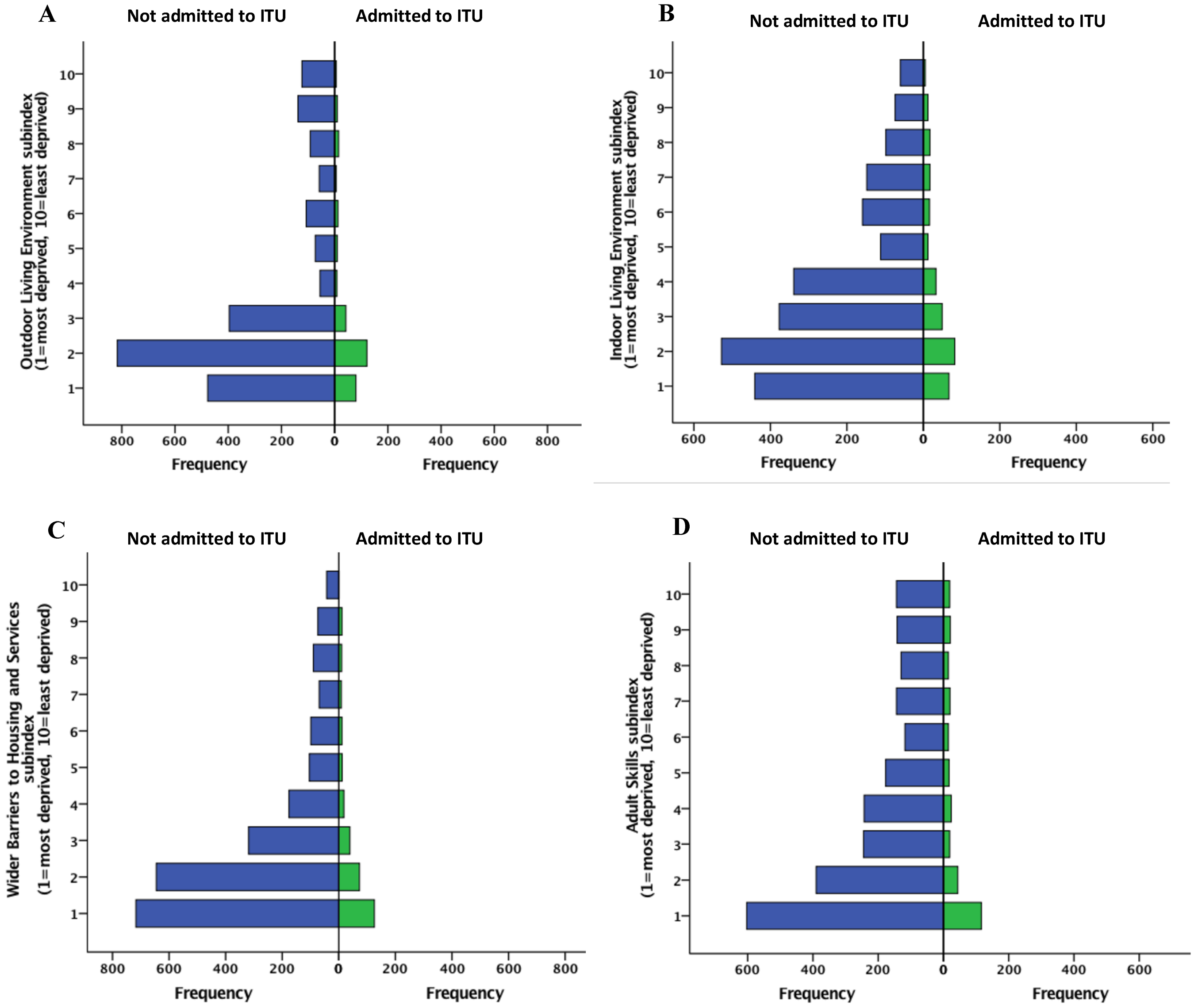

Population pyramid distributions of COVID-19 positive patients admitted to ITU by (a) Outdoor Living Environment deprivation, (b) Indoor Living Environment deprivation, (c) Wider Barriers to Housing and Services deprivation, (d) Adult Skills deprivation

Online Supplement 10

**Admission from the most deprived quintile**	**N**	**BHS deprivation**	**Wider BHS deprivation**	**LE deprivation**	**Indoor LE deprivation**	**Outdoor LE deprivation**	**Adult Skills deprivation**
Ethnic group, **n (% of ethnic group)**							
White	1917	566 (29.5)	963 (50.2)	815 (42.4)	719 (37.5)	900 (46.9)	673 (35.1)
Ethnic minorities	710	442 (62.3)	580 (81.7)	506 (71.3)	388 (54.6)	579 (81.5)	467 (65.8)
Indian	93	34 (36.6)	54 (58.1)	48 (51.6)	35 (37.6)	65 (69.9)	34 (36.6)
Pakistani	326	250 (76.7)	293 (89.9)	263 (80.7)	202 (62.0)	290 (89.0)	266 (81.6)
Caribbean	105	59 (56.2)	88 (83.8)	76 (72.4)	55 (52.4)	89 (84.8)	53 (50.5)
African	26	15 (57.7)	21 (80.8)	18 (69.2)	13 (50)	19 (73.1)	20 (76.9)
Chinese	16	4 (25.0)	13 (81.3)	10 (62.5)	9 (56.3)	11 (68.8)	7 (43.8)
Bangladeshi	11	8 (72.7)	9 (81.8)	8 (72.7)	6 (54.5)	9 (81.8)	7 (63.6)
Mixed	22	13 (59.1)	17 (77.3)	12 (54.5)	8 (36.4)	14 (63.6)	16 (72.7)
Any other ethnic group	111	59 (53.2)	85 (76.6)	71 (64.0)	60 (54.1)	82 (73.9)	64 (57.7)
Unspecified	19	13 (68.4)	18 (94.7)	14 (73.7)	11 (57.9)	16 (84.2)	13 (68.4)
**Total**	2646	1021 (38.6)	1561 (59.0)	1335 (50.5)	1118 (42.3)	1495 (56.5)	1153 (43.6)

Admissions by most deprived quintile: BHS, Wider BHS, LE, Indoor LE, Outdoor LE and Adult Skills

Online supplement 11

Univariate analyses revealed that ethnic minority COVID19 positive patients were more likely to be admitted the most deprived quintile: wider BHS [OR 4.42 (3.59-5.46); p<0.001], housing quality (indoor LE) [OR 2.01(1.69-2.39); p<0.001], air pollution (Outdoor LE) [OR 4.99(4.05-6.16); p<0.001], Adult Skills [OR 3.55(2.96-4.26); p<0.001], present with multi-lobar pneumonia [OR 2.47(2.06-2.95); p<0.001] and be admitted to ITU [OR 2.82(2.22-3.61); p<0.001] in comparison with White patients.

Online supplement 12

	ISARIC4C AUROC (95% CI)	CURB65 AUROC (95% CI)
**All patients**	0.60 (0.56-0.64)	0.62 (0.59-0.66)
**Caucasian**	0.58 (0.54-0.63)	0.67 (0.63-0.71)
**Ethnic minorities**	0.64 (0.58-0.70)	0.53 (0.46-0.59)
**Indian**	0.83 (0.73-0.93)	0.53 (0.39-0.67)
**Pakistani**	0.58 (0.47-0.69)	0.55 (0.44-0.66)
**Caribbean**	0.63 (0.49-0.78)	0.52 (0.36-0.68)
**African**	0.65 (0.34-0.96)	0.45 (0.12-0.78)
**Any other ethnic group**	0.61 (0.20-1.00)	0.61 (0.30-0.92)
**Chinese**	0.59 (0.30-0.89)	0.32 (0.01-0.63)
**Mixed**	0.63 (0.47-0.80)	0.53 (0.36-0.69)

Performance metrics for CURB65 and ISARIC4C prognostic scores by ethnic subgroup
